# Pycnogenol reduces the expression of *P. aeruginosa* T3SS and inflammatory response in NCI-H292 cells

**DOI:** 10.71150/jm.2503004

**Published:** 2025-09-19

**Authors:** Seung-Ho Kim, Da Yun Seo, Sang-Bae Han, Un-Hwan Ha, Ji-Won Park, Kyung-Seop Ahn

**Affiliations:** 1 Natural Medicine Research Center, Korea Research Institute of Bioscience and Biotechnology, Chungbuk 28116, Republic of Korea; 2 College of Life Sciences and Biotechnology, Korea University, Seoul 02841, Republic of Korea; 3 College of Pharmacy, Chungbuk National University, Chungbuk 28160, Republic of Korea; 4 Division of Practical Research, Honam National Institute of Biologucal Resources (HNIBR), Jeollanam-do 58762, Republic of Korea; 5 ELEO Inc, Cheongju-si 28160, Republic of Korea; 6 Department of Biotechnology and Bioinformatics, Korea university, Sejong 30019, Republic of Korea; 7 Advanced Research Center for Island Wildlife Biomaterials, Honam National Institute of Biological Resources (HNIBR), Jeollanam-do 58762, Republic of Korea

**Keywords:** pycnogenol, type 3 secretion system, *Pseudomonas aeruginosa*, NCI-H292, acute infection, NF-κB

## Abstract

Nosocomial infections caused by *Pseudomonas aeruginosa* (*P. aeruginosa*) have become increasingly common, particularly among immunocompromised individuals, who experience high mortality rates and prolonged treatment durations due to the limited availability of effective therapies. In this study, we screened for anti-ExoS compounds targeting *P. aeruginosa* and identified pycnogenol (PYC) as a potent inhibitor of the type III secretion system (T3SS), a major virulence mechanism responsible for the translocation of effectors such as ExoS. Using ELISA, western blotting, and real-time PCR analyses in both *P. aeruginosa* and infected H292 cells, we found that PYC significantly reduced T3SS activity. Mechanistically, PYC suppressed the transcription of T3SS-related genes by downregulating exsA expression in *P. aeruginosa*. Furthermore, pretreatment with PYC attenuated the cytotoxic effects and reduced the expression of proinflammatory cytokines, including interleukin-1β (IL-1β), interleukin-6 (IL-6), interleukin-8 (IL-8), and interleukin-18 (IL-18), in *P. aeruginosa*-infected H292 cells. These effects were associated with the inhibition of NF-κB signaling and inflammasome activation. Taken together, our findings suggest that PYC may serve as a promising therapeutic candidate against *P. aeruginosa* infections by targeting T3SS-mediated virulence and modulating host inflammatory responses.

## Introduction

Nosocomial infections caused by *Pseudomonas aeruginosa* (*P. aeruginosa*) have become increasingly prevalent in the 21st century. In 2019, the Centers for Disease Control and Prevention (CDC) identified *P. aeruginosa* as one of the multidrug-resistant super bacteria (Morales et al., 2012; Ventola, 2015). Infections with *P. aeruginosa* in immunocompromised individuals are associated with high mortality rates and prolonged treatment durations due to the lack of effective therapies. Most infections occur in hospital settings, particularly during surgical procedures when contaminated instruments come into contact with tissue (Sarges et al., 2020; Yang et al., 2022).

When *P. aeruginosa* infects a host, it utilizes type secretion systems (TSSs) located in the bacterial membrane to deliver toxins directly or indirectly into host cells, ultimately leading to cell death. TSSs are currently classified into six types based on their functions, mechanisms, and structural components (Hauser, 2009). The Type I secretion system (T1SS) exports proteases via a one-step process, whereas the Type II secretion system (T2SS) secretes various hydrolytic enzymes through a two-step mechanism involving the periplasm. The Type IV secretion system (T4SS) transfers both DNA and proteins, contributing to horizontal gene transfer and virulence. The Type V secretion system (T5SS), also called the autotransporter system, mediates the self-translocation of virulence factors. The Type VI secretion system (T6SS) delivers antibacterial toxins and virulence effectors using a contractile phage tail-like structure, facilitating both interbacterial competition and host interaction (Filloux, 2011). Among them, the Type III secretion system (T3SS) is particularly potent in inducing host cell death. The T3SS of Gram-negative bacteria, including *P. aeruginosa*, delivers exotoxins such as ExoS, ExoT, ExoY, and ExoU directly into host cells via a needle-like structure (Armentrout and Rietsch, 2016; Azimi et al., 2016). This system employs PopB/D and PcrV to form translocation pores in the host cell membrane and utilizes a long needle-like structure formed by PscF to inject effector proteins. ExoS is a bifunctional toxin with ADP-ribosyltransferase (ADPRT) activity at the C-terminus and a GTPase-activating protein (GAP) domain at the N-terminus (Mustafi et al., 2013; Saleeb et al., 2018). The ADPRT domain modifies host proteins such as Ras, RalA, Rac1, and Rabs, leading to cell death, while the GAP domain targets Rho and Rac GTPases and their downstream effectors, such as Cdc42 and ezrin/radixin/moesin proteins. These modifications disrupt the actin cytoskeleton, inhibit DNA synthesis, and interfere with key signaling pathways, ultimately compromising host cell viability (Goehring et al., 1999; Jansson et al., 2006; Rocha et al., 2003). Other T3SS exotoxins also have distinct functions. ExoU is a phospholipase A2 with strong lipolytic activity. ExoY, initially thought to function as an adenylate cyclase, was later found to act as a nucleotidyl cyclase with broad substrate specificity (Mancl et al., 2020). The expression of T3SS genes is regulated by ExsA, a transcriptional activator belonging to the LysR/AraC family. ExsA binds to the promoter (PexsC) of the exsCEBA operon and activates its transcription. Expression of exsA is further regulated by the cAMP-dependent transcription factor Vfr, which controls the intergenic region between exsB and exsA. ExsA activity is modulated by the anti-activator ExsD (Czechowska et al., 2014; Lin et al., 2021).

Host contact, low Ca^²+^ levels, or exposure to ethylene glycol-bis(β-aminoethyl ether)-N,N,N′,N′-tetraacetic acid (EGTA) triggers the secretion of the regulator ExsE from the bacterial cell. Under non-inducing conditions, ExsE binds ExsC, enabling ExsD to inhibit ExsA and repress T3SS expression. When ExsE is secreted, ExsC is freed to bind ExsD, releasing ExsA to activate T3SS gene transcription (Brutinel et al., 2010; Hauser, 2009; Zhao and Shaw, 2016).

Pycnogenol (PYC), an extract from the bark of the French maritime pine (*Pinus pinaster Aiton*), contains several flavonoids, phenolic acids, and polyphenols. PYC has been extensively studied, and its safety has been confirmed through over 40 years of clinical research. It is widely known for its broad biological activities, including antibacterial, anticancer, antiviral, antiallergic, and antioxidant effects (Lee et al., 2013; Torras et al., 2005; Wilson et al., 2010). Although it is marketed as a health supplement in approximately 50 countries, the effect of PYC on the T3SS, especially that of *P. aeruginosa*, has not yet been reported. We hypothesized that PYC, due to its established safety and wide-ranging bioactivities, might inhibit T3SS-mediated infection by *P. aeruginosa*. To test this hypothesis, we established a lung infection model using a representative *P. aeruginosa* strain and treated infected human lung epithelial (H292) cells with PYC. We analyzed T3SS activity and inflammatory responses through protein and RNA expression levels in both bacteria and host cells. Our findings demonstrate that PYC suppresses both proinflammatory cytokine production and T3SS activity, suggesting its potential as a therapeutic agent for treating T3SS-related infections.

## Materials and Methods

### Source of pycnogenol

Pycnogenol (≥ 99% purity; supplied by Healthy Origins, USA; manufactured by Horphag Research, Switzerland) was dissolved in DMSO for storage and experimental use.

### Source of *Pseudomonas aeruginosa*

*P. aeruginosa* strains were provided by Dr. Ha (Korea University). The mutant strains were derived from the wild-type strain *P. aeruginosa* K (PAK). The PAK *exoS*T::Ω (pHW0225) strain expresses ExoS tagged with a C-terminal FLAG epitope and carries insertional disruptions in both *exoS* and *exoT*. This strain was constructed by cloning the *exoS* gene into pCR2.1-TOPO, tagging it with FLAG, and inserting an Ω cassette conferring streptomycin/spectinomycin resistance. Homologous recombination into the PAK chromosome generated the exoST-deficient mutant expressing epitope-tagged ExoS (Frank et al., 1994; Kaufman et al., 2000). The PAK exsA::Ω/pHW0029 strain harbors an insertional mutation in exsA, the master transcriptional regulator of the type III secretion system (T3SS). Disruption of exsA using an Ω cassette abolished T3SS gene expression, rendering the strain secretion-deficient under T3SS-inducing conditions (Yahr et al., 1997). The PAK-ΔSTmt-pUCP18 strain was engineered to selectively secrete ExoS while lacking expression of *exoT*, *exoU*, and *exoY*. This was achieved by sequentially deleting the effector genes and complementing exoS in trans using the pUCP18 expression vector (Kaufman et al., 2000). Detailed strain is listed in Table 1. 

### Culture of *P. aeruginosa* and growth curve measurement

*P. aeruginosa* was first streaked on LB agar containing tryptone (BD Bacto^TM^ Tryptone, Cat. No. 211705, USA), NaCl (Cat. No. 19015S0350, JUNSEI, Japan), and yeast extract (Bacto^TM^ Yeast Extract, Cat. No. 212750, BD, USA), and incubated at 37°C for 24 h. A single colony was inoculated into 14 ml round-bottom tubes containing LB broth supplemented with strain-specific antibiotics (see Table 1) and cultured at 37°C with shaking at 200 rpm for 20–24 h. The resulting culture was diluted 1:1,000 into fresh LB broth supplemented with 0.5 M EGTA (Cat. No. E3889, Sigma-Aldrich, USA) and appropriate antibiotics. This subculture was incubated under the same conditions for another 20–24 h. Bacterial growth curves were analyzed by diluting cultures to an OD_600_ of 0.1, dispensing 200 μl/well into 96-well plates, treating with 20 μg/ml PYC, and monitoring OD_600_ hourly using a multifunctional plate reader (SPARK 10M, Tecan, Switzerland).

### Biofilm inhibition assay

Biofilm formation was assessed using crystal violet staining. Overnight cultures of PAK were diluted to OD_600_ = 0.1 and seeded into 96-well plates with varying concentrations of PYC (2.5, 5, 10, and 20 µg/ml). Ciprofloxacin (Ciprofloxacin, Cat. No. 17850-5G-F, Sigma, USA) was used as a positive control. After overnight incubation, non-adherent cells were removed, and wells were washed three times with distilled water. Adherent biofilms were stained with 1% crystal violet (Crystal violet, Cat. No. C3886-25G, Sigma, USA) for 1 h, washed, and air-dried. Stained biofilms were solubilized with 70% ethanol, and absorbance was measured at 585 nm using a multifunctional plate reader (SPARK 10M, Tecan, Switzerland).

### Enzyme-linked immunosorbent assay (ELISA) for measuring ExoS

PAK strains were cultured in LB containing 0.5 M EGTA, PYC (2.5–20 μg/ml), and antibiotics. Supernatants were collected by centrifugation and used to coat 96-well plates pretreated with anti-ExoS-Flag antibody (Komabiotech, Korea) diluted in carbonate-bicarbonate buffer (CBB, Cat. No. C3041-50CAP, Sigma-Aldrich, USA). Plates were incubated at 4°C overnight, blocked with 2% BSA (Cat. No. 30063-572, Gibco, New Zealand), and incubated with ExoS-containing supernatants for 2 h. Following incubation with anti-DDDDK antibody (Cat. No. ARG62342, Arigo Biolaboratories, Taiwan) and HRP-conjugated anti-mouse IgG (Cat. No. 115-035-003, Jackson ImmunoResearch, USA), colorimetric detection was performed using TMB substrate, and absorbance was measured at 450 nm.

### Real-time PCR for T3SS gene expression

Total RNA was extracted from PAK ExoS and ΔST strains using a phenol/chloroform protocol. Briefly, after coculturing bacteria with PYC (2.5, 5, 10, and 20 μg/ml) for 20–24 h, cells were pelleted and lysed with XENOSEPA_TR lysis buffer (Cat. No. S993667372TR, Xenohelix, Korea). Chloroform (1/5 vol) was added, and the aqueous RNA layer was collected. RNA was purified through ethanol precipitation and quantified using a NanoDrop spectrophotometer (NanoDrop ONE, Thermo Fisher Scientific, USA). First-strand cDNA was synthesized from 1 μg RNA using ReverTra Ace^TM^ qPCR RT Master Mix with gDNA Remover (Cat. No. FSQ-301, Toyobo, Japan) and T3SS gene-specific primers. qPCR was conducted using KAPA SYBR FAST qPCR mix (Cat. No. KK4406, KAPA Biosystems, USA). Primer sequences are provided in Table 2.

### Culture of H292 cells

H292 cells (human lung epithelial cells originating from human lung cancer tissue; H292) were purchased from *American Type Culture Collection* (ATCC, USA). H292 cells were cultured in RPMI 1640 (Cat. No. LM 011-01, WELGENE, Korea) with 10% fetal bovine serum (FBS; Invitrogen, USA), 1% antibiotics (1X penicillin-streptomycin; Invitrogen, USA) and incubated at 37℃, 5% CO_2_ incubator.

### Western blotting

H292 cells were seeded in 12-well plates and serum-starved for 1 h before PYC (20 µg/ml) treatment. After 1 h, cells were infected with PAK ExoS at MOI 100 for 1.5 h. Cells were washed with 1X DPBS containing tobramycin, lysed using a protein isolation kit, and proteins were quantified by BSA assay. Equal amounts of protein were resolved by SDS-PAGE, transferred to PVDF membranes, blocked with 5% skim milk, and probed with antibodies against IL-1β (pro-form: Cat. No. 12703S; cleaved-form: Cat. No. 83168, Cell Signaling Technology, USA), NF-κB signaling components (IKKβ: Cat. No. 8943S; p-IKKα/β: Cat. No. 2697S; p65: Cat. No. 8242S; p-p65: Cat. No. 3033S; IκBα: Cat. No. MA5-15132, Invitrogen, USA; p-IκBα: Cat. No. 2859S), β-actin (Cat. No. SC-4778, Santa Cruz, USA), NLRP3 (Cat. No. 15182S), and Caspase-1 (Cat. No. 3866S). HRP-conjugated anti-rabbit secondary antibodies were used (1:5000), and detection was performed using ECL solution (Pierce^TM^ ECL Western Blotting Substrate, Cat. No. 32106, Thermo Fisher Scientific, USA).

### ELISA for measuring of pro-inflammatory cytokines

H292 cells were seeded in 12-well plates at a density of 5 × 10^5^ cells/ml and incubated at 37°C in a 5% CO_2_ incubator for 24 h. The cells were then serum-starved using RPMI 1640 medium without FBS for 2 h, followed by treatment with PYC at concentrations of 2.5, 5, 10, and 20 μg/ml for 1 h. After treatment, the cells were infected with PAK ExoS at a multiplicity of infection (MOI) of 100 for 1.5 h. To remove extracellular bacteria, cells were washed three times with 1% DPBS containing tobramycin. Subsequently, fresh RPMI 1640 medium containing 0.1% FBS was added, and cells were further incubated for 4 h. After the final incubation, cell culture supernatants were harvested and stored at –80°C until analysis. ELISA was performed according to the manufacturer’s instructions using commercial kits for human IL-1β (Cat. No. 557953, BD Biosciences, USA), IL-6 (Cat. No. 555220, BD Biosciences, USA), IL-8 (Cat. No. DY208, R&D Systems, USA), and IL-18 (Cat. No. DY318-05, R&D Systems, USA). Absorbance was measured at 450 nm using a multifunctional plate reader (SPARK 10M, Tecan, Switzerland).

### Real-time PCR analysis for measuring pro-inflammatory cytokines

RNA for inflammatory cytokines in H292 cells was obtained by incubating H292 cells with 5 × 10^5^ cells/ml in 12-well plates. First, the cells were seeded and incubated for 1 day in a 37℃ incubator, and then the cells were starved using non-FBS RPMI 1640 media for 2 h. The cells were infected with PAK ExoS at an MOI of 100 for 1.5 h after treatment with PYC (2.5, 5, 10, and 20 μg/ml) for 1 h, and then the bacteria were eliminated with 1% DPBS, including tobramycin. Next, total RNA lysis buffer (XENOSEPA_TR lysis buffer, Cat. No. S993667372TR, Xenohelix, Korea) was added to the cells, and they were centrifuged. Next, approximately 1/5 volume of chloroform was added to obtain an RNA layer, and RNA was extracted during the wash procedures and quantified at 1 μg/µl using a Nanodrop (NanoDrop ONE, Thermo Fisher, USA). A cDNA synthesis kit (ReverTra Ace^TM^ qPCR RT Master Mix with gDNA Remover, Cat. No. FSQ-301, Toyobo, Japan) was used to synthesize cDNA using primers for each gene. After that, an SYBR green system was used to detect the amplified genes in the cDNA in real-time. Detailed information on the sequence is noted in Table 2.

### Immunocytochemistry

H292 cells were seeded in 8-well chamber slides at 3 × 10^4^ cells/well and incubated at 37°C for 24 h. Cells were serum-starved for 1 h, treated with 20 μg/ml PYC for 1 h, and infected with PAK ExoS at MOI 100 for 1 h. Following three washes with 1% DPBS containing tobramycin, cells were fixed with 4% formaldehyde (Cat. No. BBC0150, BBC Biochemical, USA) for 5 min at room temperature. Permeabilization was performed with 0.1% Triton X-100 for 15 min at 4°C. After blocking with 3% BSA for 1 h, cells were incubated with primary antibodies (1:500 dilution in 3% BSA) overnight at 4°C, followed by Alexa Fluor 488-conjugated secondary antibodies (1:500) for 1 h at room temperature in the dark. Nuclei were counterstained with Hoechst 33342 (Cat. No. H3570, Thermo Fisher Scientific, USA) for 1 h. After final washes, slides were mounted with 10 μl mounting solution and covered with glass coverslips. Fluorescence images were acquired using an Axio Observer Z1 microscope (Zeiss, Germany).

### Statistical analysis

Data are presented as mean ± standard deviation (SD). Statistical significance was evaluated using ordinary one-way ANOVA followed by Dunnett’s multiple comparisons test, with a p-value < 0.05 considered statistically significant. For datasets comparing only two groups, such as normalized protein band intensities in Figs. 5 and 6, a two-tailed unpaired Student’s t-test was employed.

## Results

### PYC reduces biofilm formation and secretion of ExoS in *P. aeruginosa*

**Fig. 1.**
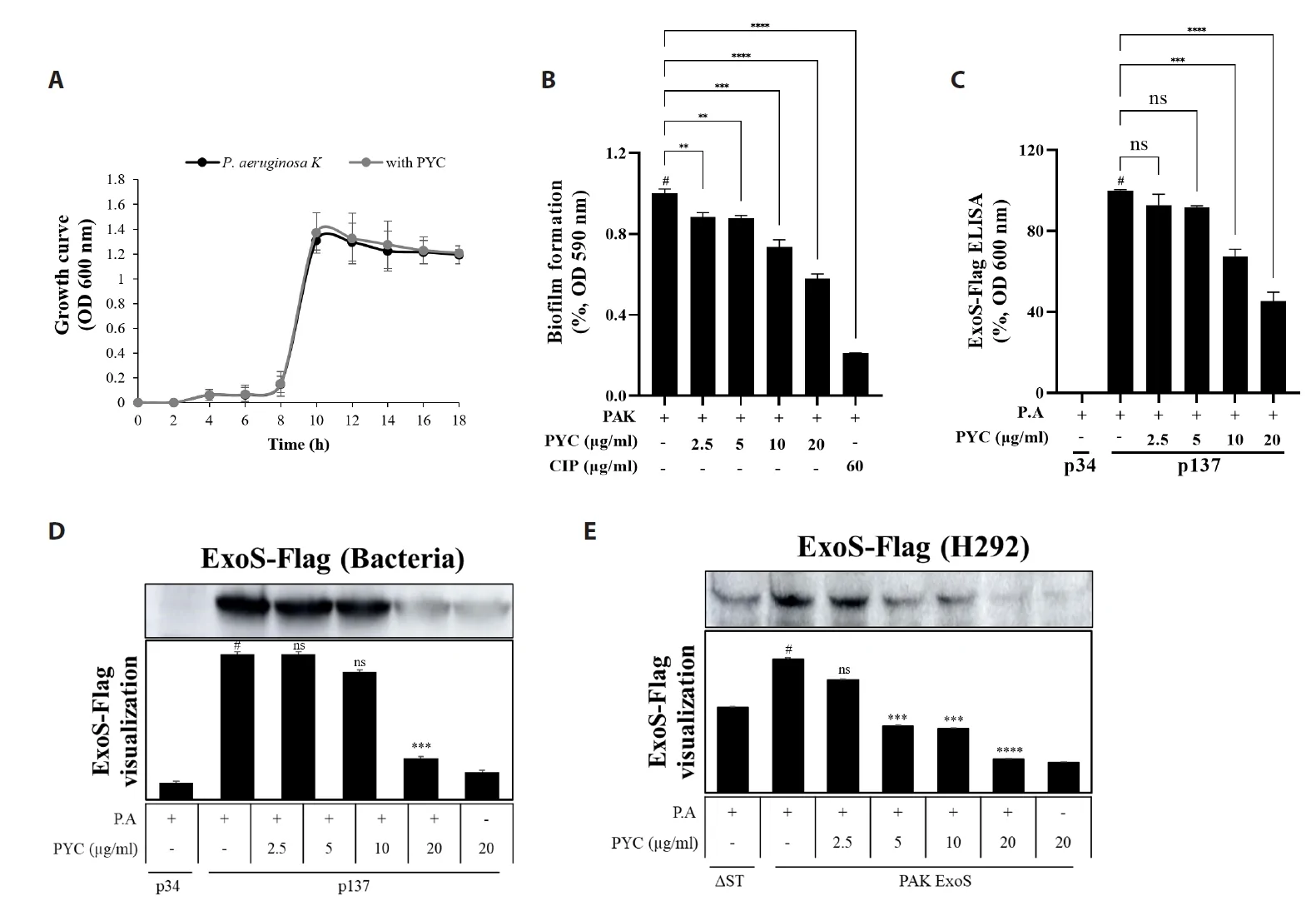
Effect of PYC on *P. aeruginosa*. All experiments were conducted in triplicate. Various aspects of *P. aeruginosa* behavior were examined in the presence of PYC: growth curve of *P. aeruginosa* (A), biofilm formation (B), secretion of ExoS (C), level of ExoS-Flag in bacteria (D), and delivered level of ExoS-Flag with infected H292 (E). The control group was exposed to 60 μg/ml ciprofloxacin (CIP). The type III secretion system (T3SS) was upregulated by co-adding 0.5 M EGTA during bacterial culturing. Anti-ExoS-Flag ELISA and ExoS-Flag of western blot were performed using the p137 or PAK ExoS strain, which secretes ExoS-Flag, and the T3SS-defective p34 and ΔST strain. The positive control is denoted as ‘#’. Statistical significance was determined by one-way ANOVA followed by Dunnett’s post hoc test. p < 0.05, p < 0.01, p < 0.001, p < 0.0001 vs. control.

Pycnogenol (PYC) is known for its anticancer, antioxidant, and anti-inflammatory properties; however, its potential to modulate the virulence of *P. aeruginosa* has not been fully explored. To investigate this, we examined the effects of PYC on bacterial growth, biofilm formation, and secretion of ExoS, a key effector protein of the type III secretion system (T3SS). First, we compared the growth kinetics of *P. aeruginosa* in the presence or absence of 20 µg/ml PYC (Fig. 1A). No significant differences were observed, indicating that PYC does not affect bacterial proliferation. In contrast, biofilm production was significantly reduced in the presence of PYC, suggesting that PYC disrupts a major mechanism by which *P. aeruginosa* evades host immune responses (Fig. 1B). To assess the impact of PYC on T3SS-associated virulence, we measured ExoS secretion using an anti-ExoS enzyme-linked immunosorbent assay (ELISA). When the p137 strain was treated with increasing concentrations of PYC, a dose-dependent reduction in secreted ExoS was observed (Fig. 1C). The p137 and p34 strains were employed to distinguish T3SS-specific effects. p137 expresses FLAG-tagged ExoS, while p34 lacks a functional T3SS due to exsA disruption. Western blot analysis further confirmed a concentration-dependent decrease in ExoS-Flag levels in the culture supernatant of the p137 strain following PYC treatment (Fig. 1D). Similarly, a reduction in ExoS-Flag was detected in H292 cells infected with the PAK ExoS (Fig. 1E). Collectively, these findings indicate that PYC inhibits both biofilm formation and ExoS secretion in *P. aeruginosa*. These effects may reflect a broader regulatory impact on T3SS-associated virulence factors.

### The *P. aeruginosa* T3SS is regulated by PYC through inhibition of *ExsA* mRNA

**Fig. 2.**
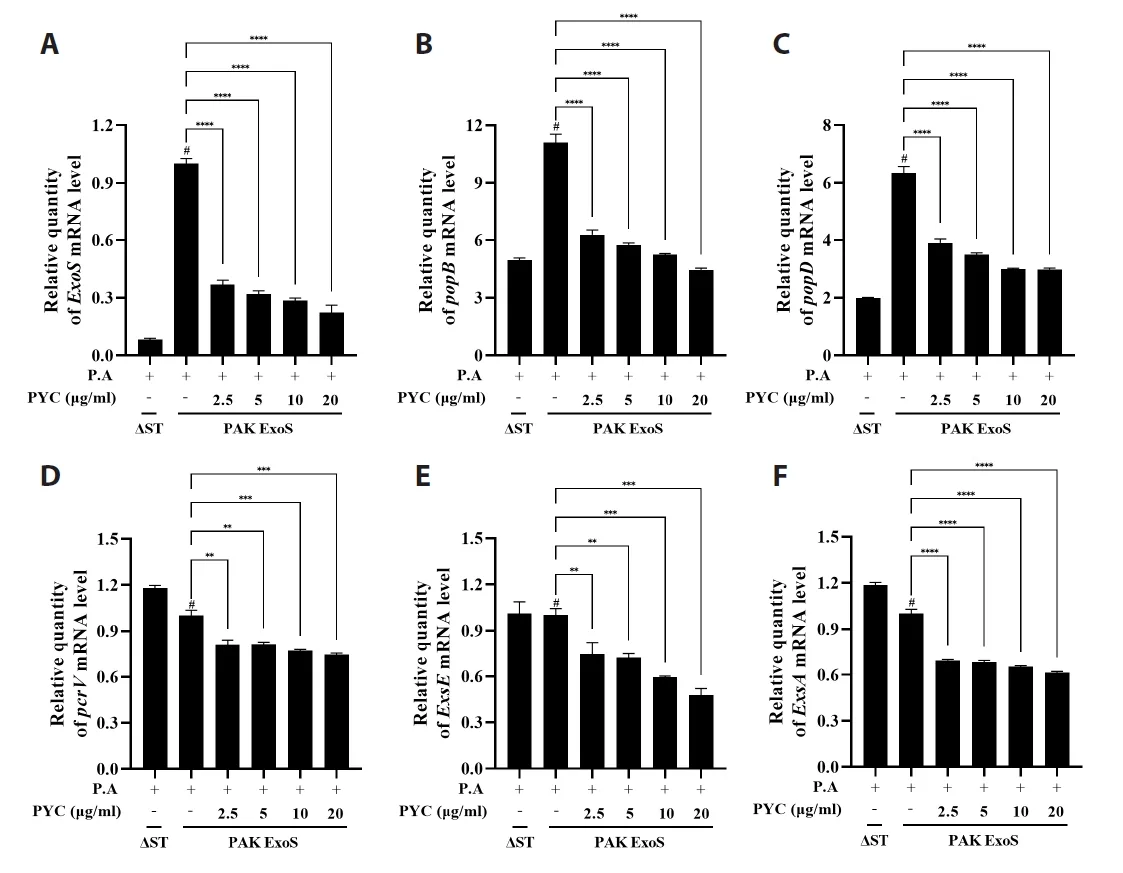
Inhibition of *P. aeruginosa* T3SS expression by PYC. The experimental strains included the PAK ExoS strain, engineered to exclusively secrete the ExoS exotoxin, and the ∆ST strain, lacking the gene encoding exotoxin S/T. PAK ExoS were cultured in the presence of PYC. The measured mRNA levels included transcripts for ExoS, the toxin delivered into the host cell (A); *popB*, responsible for forming a pore in the host cell membrane (B); *popD*, which interacts with *popB* (C); bacterial mRNA of *pcrV*, a conduit facilitating the transport of internal ExoS to the host (D); mRNA of ExsE, a regulator of T3SS activity (E); and mRNA of ExsA, a master regulator governing the overall T3SS (F). The positive control is denoted as ‘#’. Statistical significance was determined by one-way ANOVA followed by Dunnett’s post hoc test. p < 0.05, p < 0.01, p < 0.001, p < 0.0001 vs. control.

To further evaluate the impact of PYC on T3SS-related components, we performed real-time PCR analysis. Consistent with our ELISA results, *exoS* expression was reduced in a dose-dependent manner following PYC treatment (Fig. 2A). In addition, the expression levels of *popB* and *popD*, which encode translocator proteins responsible for pore formation in the host membrane, were significantly decreased at PYC concentrations of 10 and 20 μg/ml (Fig. 2B and 2C). Expression of *pcrV*, a structural component involved in the translocation of ExoS, was also reduced at 20 μg/ml PYC (Fig. 2D). Similarly, the expression of *exsE*, which regulates the availability of the ExsA transcription factor, declined in a concentration-dependent manner (Fig. 2E). Importantly, we observed that the expression of *exsA*, a central regulator of T3SS gene transcription, was significantly suppressed by PYC (Fig. 2F). Given the key role of ExsA in activating downstream T3SS effectors, this result suggests that PYC-mediated inhibition of exsA may contribute to the broader suppression of T3SS activity. In summary, our qPCR results demonstrate that PYC downregulates the expression of multiple T3SS-associated genes, including *exoS*, *popB/D*, *pcrV*, *exsE*, and *exsA*. These findings support the notion that PYC inhibits ExoS secretion and overall T3SS activity by targeting ExsA-dependent transcriptional regulation.

### PYC inhibits the expression and production of increased inflammatory cytokines in infected H292 cells

**Fig. 3.**
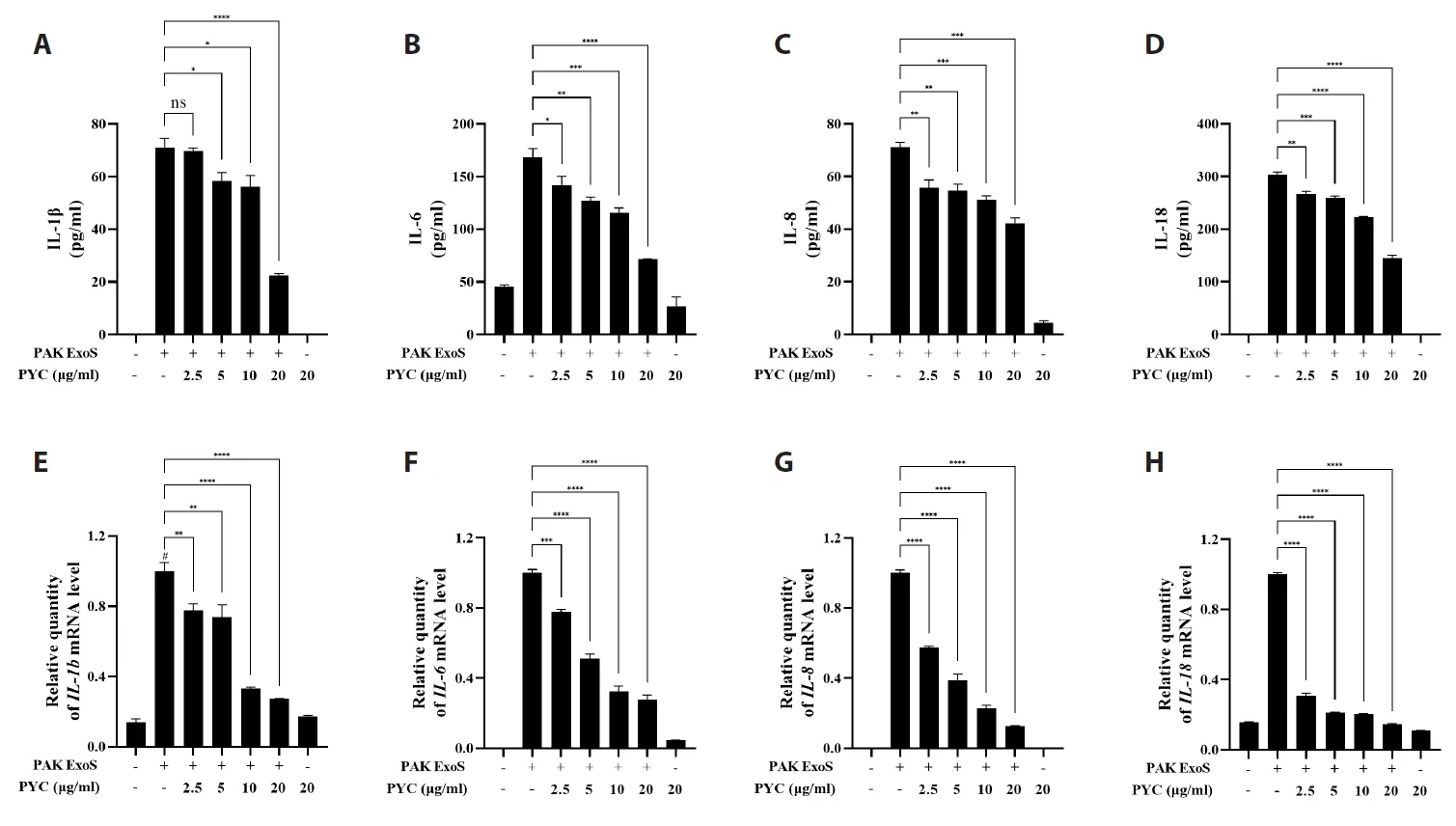
Expression and production levels of inflammatory cytokines inhibited by PYC in H292 cells. Changes in inflammatory cytokines induced by *P. aeruginosa* were measured using ELISA and real-time PCR. The PAK ExoS was used. To upregulate the T3SS in culture, 0.5 M EGTA was used, and PYC was added to H292 cells before infection. The infection was carried out at an MOI of 100. The production levels of IL-1β (A), IL-6 (B), IL-8 (C), IL-18 (D), and the mRNA levels of IL-1β (E), IL-6 (F), IL-8 (G), and IL-18 (H) were measured. The positive control is denoted as ‘#’. Statistical significance was determined by one-way ANOVA followed by Dunnett’s post hoc test. p < 0.05, p < 0.01, p < 0.001, p < 0.0001 vs. control.

During *P. aeruginosa* lung infection, sustained production of proinflammatory cytokines is induced, primarily in response to bacterial components such as lipopolysaccharide (LPS), flagella, and ExoS. To evaluate the effect of PYC on inflammation, we examined cytokine production in H292 cells infected with the PAK ExoS strain, which secretes only ExoS without other effectors such as ExoY, ExoU, or ExoT. Treatment with PYC led to a concentration-dependent reduction in the levels of IL-1β, IL-6, IL-8, and IL-18, with 20 μg/ml PYC showing the most pronounced effect (Fig. 3A–3D). These results indicate that PYC attenuates the heightened inflammatory response associated with *P. aeruginosa* infection. To determine whether the decrease in cytokine production was due to transcriptional regulation, we performed real-time PCR to assess mRNA expression levels. Interestingly, the suppressive effects of PYC on cytokine mRNA expression were even more pronounced than the reductions observed at the protein level (Fig. 3E–3H). This suggests that PYC primarily exerts its anti-inflammatory effects through transcriptional inhibition of proinflammatory cytokines. Taken together, our findings demonstrate that PYC mitigates the excessive inflammatory response triggered by *P. aeruginosa* infection by downregulating both the transcription and secretion of key proinflammatory cytokines. These results highlight the potential of PYC as a therapeutic agent for modulating host inflammatory responses during bacterial lung infections.

### PYC regulates IL-1β by attenuating NF-κB and the inflammasome, which are activated in infected H292 cells

**Fig. 4.**
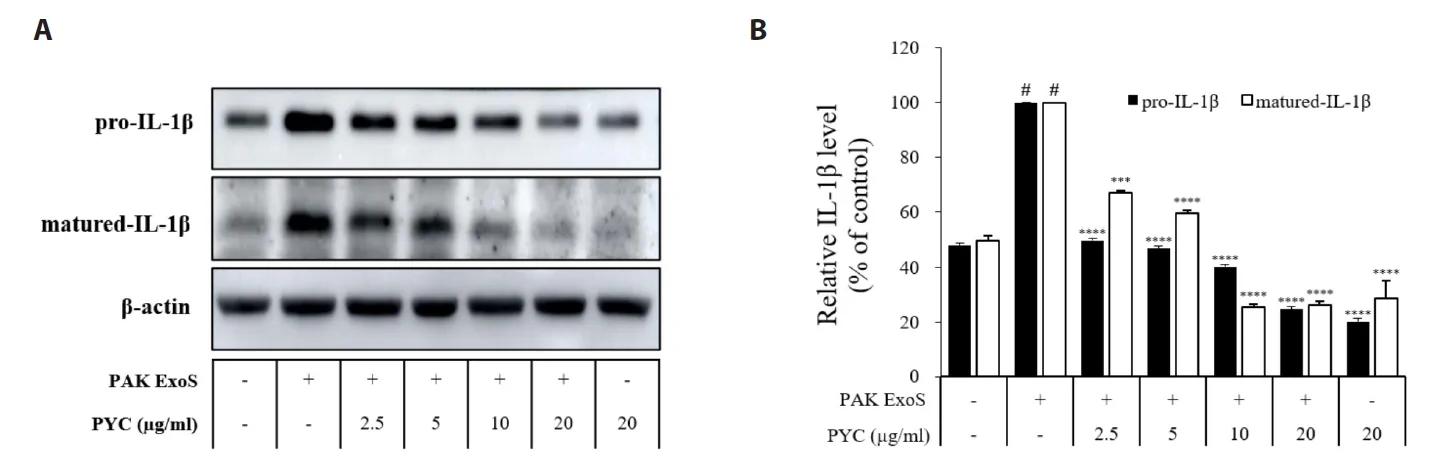
Levels of pro-form and mature-form IL-1β in infected H292 cells treated with PYC. To examine the levels of IL-1β in infected H292 cells, the cells were infected with PAK ExoS at an MOI of 100. PYC treatment was performed before infection. A protein band of pro-form and mature-form IL-1β (A), visualization graph of the protein bands for IL-1β (B). ImageJ software was utilized for the visualization of each protein band. The positive control is denoted as ‘#’. Statistical significance was determined by one-way ANOVA followed by Dunnett’s post hoc test. p < 0.05, p < 0.01, p < 0.001, p < 0.0001 vs. control.

**Fig. 5.**
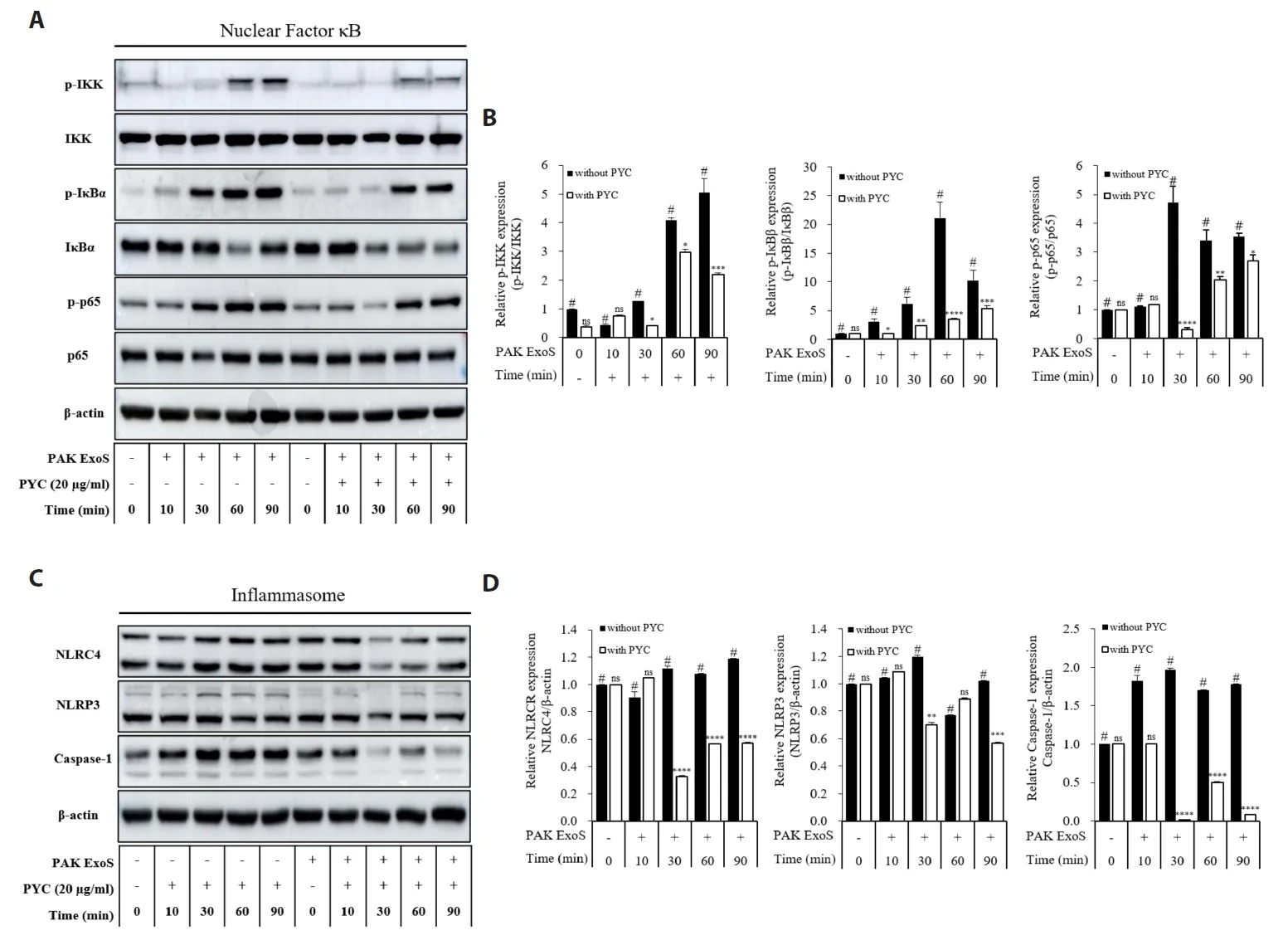
NF-κB and inflammasome proteins in infected H292 cells treated with PYC. H292 cells were treated with 20 µg/ml PYC and subsequently infected with PAK ExoS at an MOI of 100 for 10, 30, 60, and 90 min. A protein band of NF-κB pathway (A), visualization graph of the protein bands for NF-κB (B), a protein band of inflammasome (C), and visualization graph of the protein bands for inflammasome (D). Image J software was utilized for the visualization of each protein band. The positive control is denoted as ‘#’. Statistical significance was determined using a two-tailed Student’s t-test for comparisons between two groups. p-values < 0.05 were considered statistically significant (^*^p < 0.05, ^**^p < 0.01).

During *P. aeruginosa* infection, interleukin-1β (IL-1β) plays a critical role in recruiting neutrophils to the site of infection. IL-1β is produced as an inactive pro-form that requires cleavage into its mature, active form by inflammasome components. While previous ELISA and real-time PCR experiments confirmed that PYC reduces IL-1β levels, it remained unclear whether PYC affects both forms of IL-1β. To address this, we performed western blot analysis to detect the pro- and mature forms of IL-1β. The results showed that treatment with PYC led to a concentration-dependent decrease in both forms of IL-1β (Fig. 4A), suggesting that PYC not only inhibits IL-1β production but also interferes with its maturation process. To further elucidate the mechanism, H292 cells were infected with *P. aeruginosa* in the presence of 20 μg/ml PYC, and samples were collected at multiple time points post-infection. Phosphorylation of the transcription factor p65 was reduced as early as 30 min after infection in PYC-treated cells, along with decreased phosphorylation of upstream regulators IκBα and IKK (Fig. 5A). However, the total level of IκBα did not immediately recover despite reduced phosphorylation. To determine whether this was due to continued degradation, we monitored IκBα levels over longer infection periods and observed a delayed recovery (Fig. S2). We next examined inflammasome activation, which mediates IL-1β maturation. Western blot analysis revealed that levels of caspase-1 and NLRP3 were significantly reduced in PYC-treated cells compared to untreated controls (Fig. 5C). These findings indicate that PYC inhibits both the transcriptional induction and inflammasome-mediated processing of IL-1β. In summary, our data demonstrate that PYC attenuates IL-1β-mediated inflammatory responses by targeting both NF-κB signaling and inflammasome activation. These results provide further insight into the molecular mechanisms by which PYC suppresses IL-1β production and activity during *P. aeruginosa* infection.

### PYC attenuates the inflammatory response in infected H292 cells by inhibiting p65 in the cytosol and nucleus

**Fig. 6.**
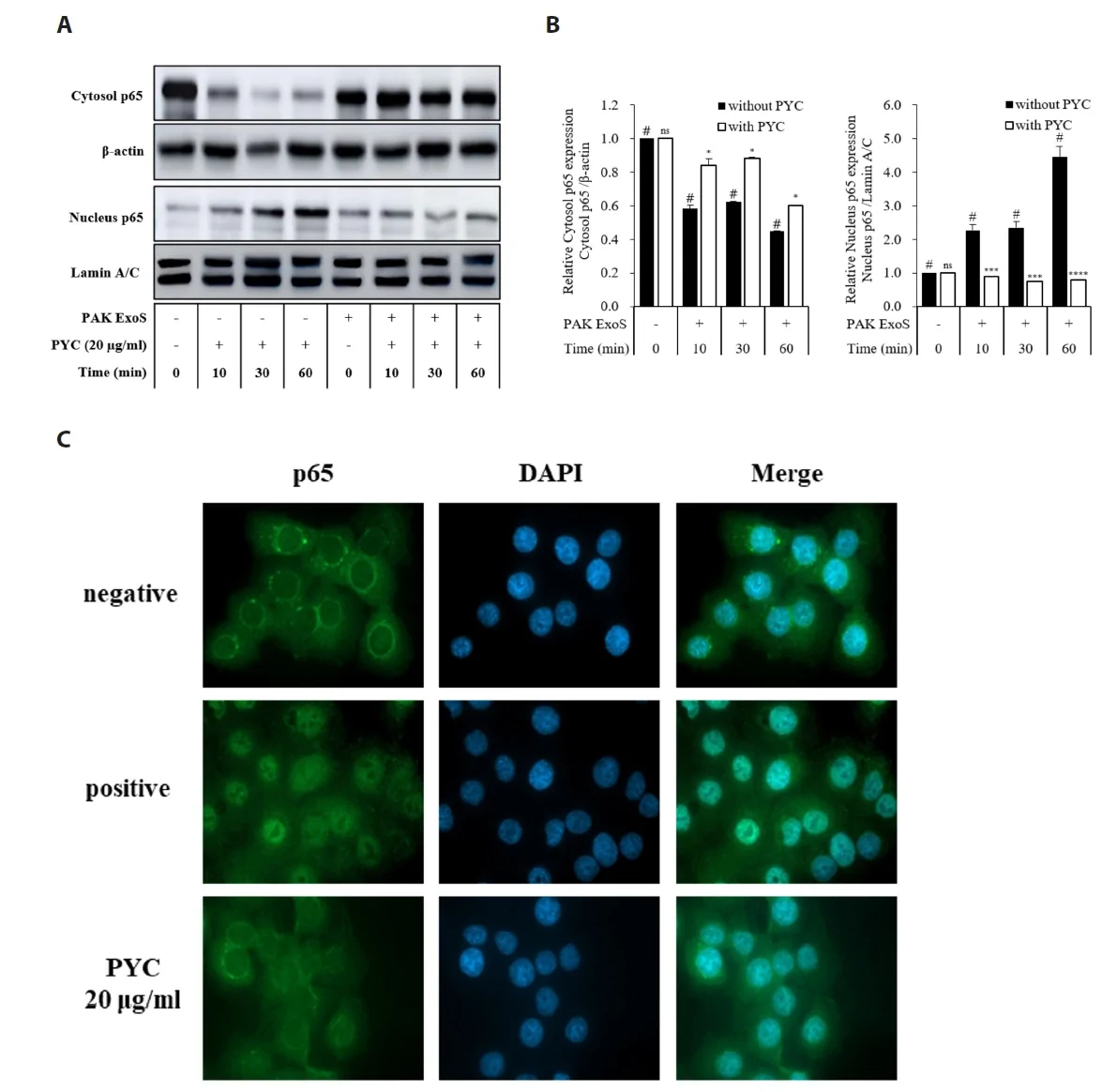
Western blot and immunocytochemistry to confirm the translocation of p65. H292 cells were treated with 20 µg/ml PYC and subsequently infected with PAK ExoS at an MOI of 100 for 10, 30, 60, and 90 min. Then, a western blot was performed after the protein fractured the cytosol and nucleus. A protein band of p65 in cytosol and nucleus (A), visualization graph of the protein bands for translocation of p65 (B). Immunostaining of p65 in H292 cells (C), the scale bar of the image indicates 20 µm. Statistical significance was determined using a two-tailed Student’s t-test for comparisons between two groups. p-values < 0.05 were considered statistically significant (^*^p < 0.05, ^**^p < 0.01).

We observed a decrease in IL-1β levels due to the inhibitory effect of PYC, as demonstrated by our previous experimental results. To further validate this effect, we investigated the translocation of p65 by fractionating cytoplasmic and nuclear proteins in infected H292 cells over time at an MOI of 100. Upon treatment with 20 μg/ml PYC, cytoplasmic levels of p65 progressively increased, whereas nuclear levels decreased over time (Fig. 6A). Furthermore, fluorescence staining revealed that PYC treatment led to a reduced nuclear translocation of p65 during *P. aeruginosa* infection, as compared across different infection durations (Fig. 6C). These findings strongly suggest that PYC inhibits the nuclear translocation of p65, consistent with our initial hypothesis and supporting a potential mechanism underlying the observed reduction in IL-1β levels.

### PYC alleviates inflammation by inhibiting NF-κB signaling and the inflammasome in infected H292 cells

**Fig. 7.**
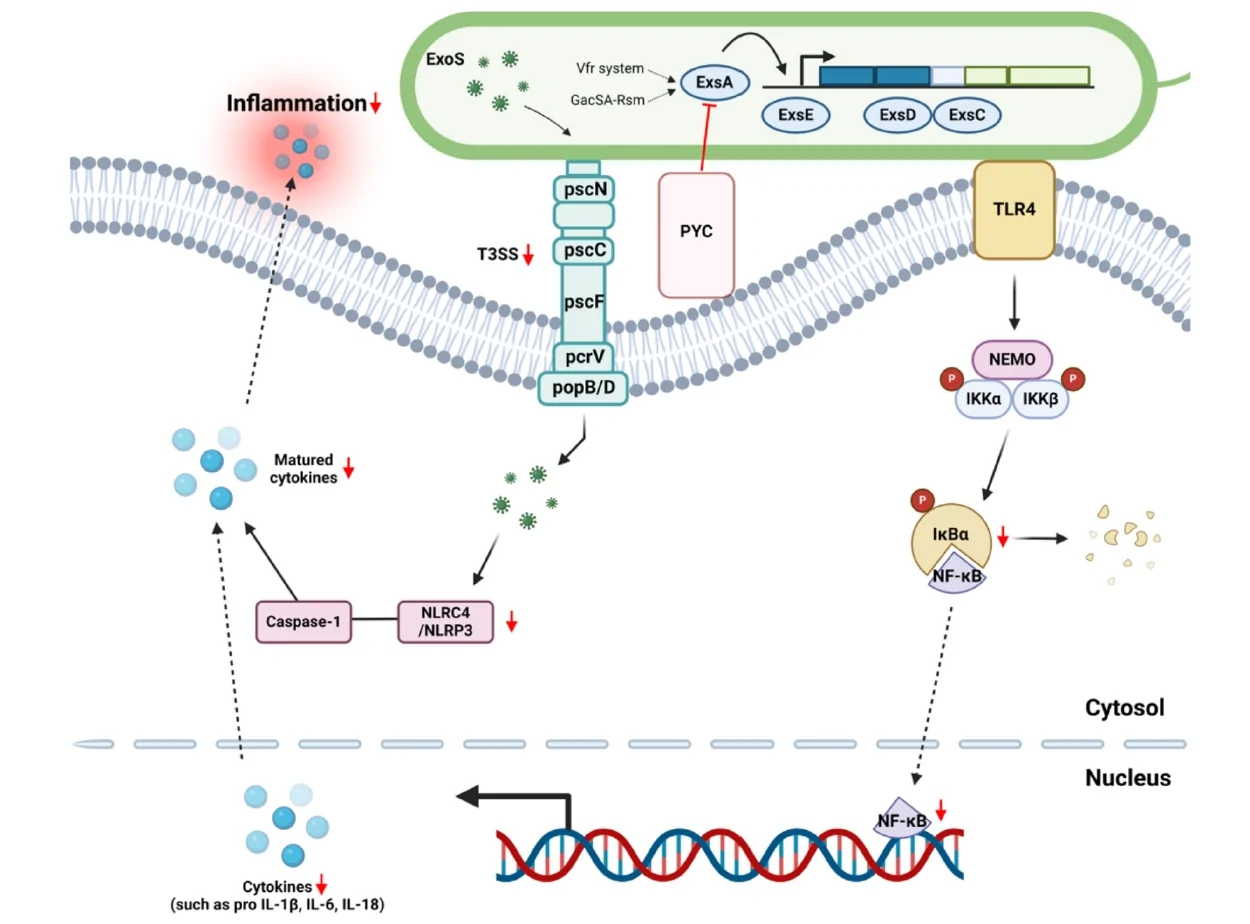
The effect of pycnogenol on NF-κB signaling, inflammasome activity, and IL-1β production in H292 cells infected with *P. aeurignosa*. This figure was made using a biorender and was indicated with a signaling pathway when infected with *P. aeruginosa* in H292 cells. PYC can attenuate the expression of T3SS and inflammation in H292 cells. The red arrows indicate the effect of PYC.

In our previous experiments, we investigated the role of PYC in modulating the inflammatory response of H292 cells during *P. aeruginosa* infection. The NF-κB signaling pathway is known to be activated by bacterial components such as lipopolysaccharide (LPS) and flagellin, leading to the production of pro-IL-1β. In this study, we found that PYC regulates NF-κB activation by inhibiting the phosphorylation of IKK, which subsequently downregulates the expression of pro-IL-1β. During infection, *P. aeruginosa* delivers various virulence factors, including FliC and needle complex proteins, into host cells, which can activate caspase-1. However, treatment of H292 cells with PYC was found to modulate ExsA, resulting in reduced ExoS secretion and, consequently, a decrease in mature IL-1β levels (Fig. 6). These findings indicate that PYC inhibits T3SS-mediated pathogenicity by interfering with key signaling pathways involved in the *P. aeruginosa*-induced inflammatory response. Moreover, PYC also inhibited inflammasome components, such as caspase-1 and NLRP3, which are responsible for cytokine maturation and secretion. Collectively, these results suggest that PYC exerts its anti-inflammatory effects by targeting multiple steps in the host inflammatory cascade and may serve as a promising therapeutic candidate for controlling *P. aeruginosa*-induced pathogenicity (Fig. 7).

## Discussion

Lung infections caused by *P. aeruginosa* are becoming increasingly prevalent. Despite the rising incidence, effective treatment remains challenging due to the bacterium’s strong antibiotic resistance and its ability to evade host immune responses. This study aimed to identify a compound that specifically inhibits the type III secretion system (T3SS) in *P. aeruginosa* and to propose a novel therapeutic strategy. Pycnogenol (PYC) is a natural compound that is generally considered safe and well-tolerated, with no reported toxicity at recommended doses. Its safety and health benefits have been supported by over 40 years of clinical research. While adverse effects such as gastrointestinal discomfort, headache, or allergic reactions are rare, they may occur in sensitive individuals. In our study, we established appropriate PYC concentrations for use in bacterial and cell culture models through bacterial growth curve and cytotoxicity assays (Figs. 1A and S1). Treatment with 20 μg/ml PYC did not affect bacterial growth across all tested strains, whereas concentrations above 80 μg/ml significantly inhibited growth (data not shown), consistent with prior reports on PYC’s antimicrobial activity. Importantly, PYC inhibited biofilm formation and the extracellular secretion of ExoS-Flag (Fig. 1B and 1C), suggesting that it modulates *P. aeruginosa* virulence pathways. Biofilm formation—mediated by T6SS in chronic infections—and ExoS secretion—governed by T3SS in acute infections were both suppressed. As *P. aeruginosa* dynamically regulates T3SS and T6SS via phenotypic switching during infection, PYC may interfere with this regulatory transition (Chen et al., 2020; Zhu et al., 2016). At the molecular level, PYC suppressed the expression of ExsA, a master regulator of T3SS and member of the LysR/AraC family, resulting in reduced expression of downstream T3SS effectors (Fig. 2A–2F). Since ExsA expression is typically induced under low calcium conditions and is regulated by the Vfr-PexsA/PexsC system, further investigation is needed to determine whether PYC modulates this regulatory axis. Although we observed that PYC significantly reduced exsA transcription, the upstream regulatory mechanisms involved in this process remain unclear. In particular, the Vfr–cAMP axis, which modulates exsA promoter activity via PexsA/C, was not addressed in this study. Future studies employing promoter–reporter assays, Vfr-binding analysis, and ligand–protein interaction techniques such as SPR or BLI will be necessary to determine whether PYC acts via this known pathway or targets a novel regulator.

Infected H292 cells were used to evaluate PYC’s anti-inflammatory effects. Cytokine analysis in cells infected with the ExoS-only PAK strain (PAK ExoS) revealed that PYC significantly reduced transcription and secretion of IL-1β, IL-6, IL-8, and IL-18 in a dose-dependent manner (Fig. 3). IL-1β is known to play a central role in pneumonia pathogenesis via neutrophil recruitment (Cohen and Prince, 2013; Hardy et al., 2021; Karmakar et al., 2012), while IL-6 and IL-8 are key regulators of early inflammatory responses (Bickel, 1993; Tanaka et al., 2014), and IL-18 contributes to host defense (Ihim et al., 2022). These findings suggest that PYC broadly suppresses proinflammatory cytokine responses in infected epithelial cells. IL-1β production is tightly regulated by Toll-like receptor signaling and inflammasome activation. LPS and flagellin stimulate TLR4 and TLR5, respectively, leading to NF-κB activation through the IKK/IκBα/p65 axis (Liu et al., 2017). NF-κB activation drives the transcription of pro-IL-1β, a 34-kDa cytosolic precursor that requires proteolytic cleavage by caspase-1 for maturation. Caspase-1, activated by inflammasome complexes such as NLRP3 and NLRC4 in response to bacterial components like FliC and T3SS needles, cleaves IL-1β to its mature 17-kDa form (Ahn et al., 2023; Galliher-Beckley et al., 2013). In our study, PYC treatment reduced both pro- and mature forms of IL-1β in infected H292 cells, indicating inhibition of both transcription and maturation (Fig. 4A and 4B). To explore the signaling mechanisms underlying this effect, we examined NF-κB and inflammasome pathways over time. PYC suppressed IKK phosphorylation and p65 nuclear translocation (Fig. 5A), while also reducing expression of inflammasome components such as NLRP3 and NLRC4 (Fig. 5C). The sharp reduction in p-p65 levels at 30 min following PYC treatment likely reflects upstream inhibition of the IKK/IκBα axis, as shown by suppressed phosphorylation of IKK and IκBα at the same time point. This would block IκBα degradation and prevent p65 nuclear translocation and activation. Interestingly, p-p65 levels partially recovered at 60 and 90 min, albeit remaining lower than in untreated controls, suggesting a transient but strong early-phase inhibition that may be followed by cellular compensation or reduced PYC activity over time. Although IκBα levels remained low during early infection despite reduced IKK activity, recovery of IκBα at 120 min post-infection (Fig. S2) suggests delayed synthesis or degradation mediated by bacterial signals such as LPS or quorum-sensing molecules like C12 (Kravchenko et al., 2008; Wang et al., 2011). Caspase-1 activity was also significantly reduced by PYC, although it remains unclear whether this is due to decreased ExoS delivery or a direct effect on inflammasome assembly. Additionally, p65 nuclear localization was suppressed by PYC, as confirmed by both western blot and immunofluorescence (Fig. 6A–6C). These findings demonstrate that PYC interferes with T3SS-mediated delivery of ExoS as well as host inflammatory signaling. In conclusion, our study provides evidence that PYC targets multiple levels of *P. aeruginosa* virulence and host immune activation, including inhibition of T3SS transcription (via ExsA), NF-κB signaling, and inflammasome-mediated IL-1β maturation. These results support the potential use of PYC as a T3SS-specific anti-virulence agent for mitigating acute lung inflammation caused by *P. aeruginosa* (Fig. 7).

## Conclusion

In conclusion, our study presents a novel therapeutic strategy against *Pseudomonas aeruginosa* infections. We have demonstrated that Pycnogenol (PYC) regulates the transcription of the type III secretion system (T3SS) in *P. aeruginosa*, impacting associated inflammatory responses, particularly in acute lung inflammation. PYC exhibited inhibitory effects on *P. aeruginosa* growth, biofilm formation, and the extracellular release of ExoS-Flag protein, directly linked to the suppression of T3SS. Furthermore, our research confirmed that PYC reduces inflammation responses induced by *P. aeruginosa* in H292 cells. Notably, the transcription and production levels of key cytokines such as IL-1β, IL-6, IL-8, and IL-18 were significantly reduced following PYC treatment. These findings suggest that PYC mitigates inflammation by modulating the NF-κB signaling pathway and inhibiting the maturation of IL-1β. Therefore, PYC holds potential as an effective T3SS-specific therapeutic agent in regulating *P. aeruginosa* infections and their associated inflammatory responses. However, further research is required to fully understand the precise mechanisms of action and long-term effects of PYC. Such studies will not only contribute to the treatment of *P. aeruginosa* infections but also to the development of broader therapeutic strategies against other pathogenic microorganisms. This conclusion succinctly summarizes the key findings and implications of the research, highlighting the potential role of PYC and the need for further investigation.

## Figures and Tables

**Table 1.** List of all strains and plasmids used in this study

Strains	Description	Antibiotics	Source
*P. aeruginosa K* (PAK)	Clinical isolate of Wild-type invasive strain	-	From Dr. Ha (Korea University)
PAK exsA::Ω/pHW0029 (p34; negative control)	PAK with chromosonal disruption of the *exsA* loci with Ω cassette	Sp^200^, Sm^200^, Gm^200^, Cb^150^	From Dr. Ha
PAK exoST::Ω/pHW0225 (p137)	PAK ExoST double mutated exoS-FLAG	Sp^200^, Gm^200^, Cb^150^	From Dr. Ha
PAK ΔST	Double effector mutant; functional needle and translocon apparatus without known effectors	-	From Dr. Ha
PAK ΔST/pUCP18 (PAK ExoS)	PAK ΔST with exoS of pUCP18	Cb^150^	From Dr. Park

* Summary of *P. aeruginosa* strains used in this study. Detailed genetic backgrounds and construction methods are described in the cited references.

**Table 2.** List of all primers used in this study

*P. aeruginosa* primers	Sense (5’→3’)	Anti-sense (5’→3’)
16S	CAA AAC TAC TGA GCT AGA GTA CG	GCC ACT GGT GTT CCT TCC TA
*ExoS*	CAG GCT GAA CAG GTA GTG AA	TTC AGG GAG GTG GAG AGA TA
*popB*	GCG CTT CGA CGC TGT TGT	TTC TTC CGA CTC CCT GAT CTT CT
*popD*	GAA GAC CCT GCA GAA GAA CA	ACC TTG CCG ACG ATC TTG
*pcrV*	GAT CGA CGC TGG CGG TAT	TCA TCG CTG AGG CCC TTG
*ExsE*	TGC TGT TCG ACG AAC AGG TG	ATC GTT TGC ATC GCT CCC TG
*ExsA*	AAG GAG CCA AAT CTC TTG	CTT GTT TAC CCT GTA TTC G
Human primers		
*GAPDH*	GCA GGG GGG AGC CAA AAG GG	TGC CAG CCC CAG CGT CAA AG
*IL-1β*	GG ACA AGC TGA GGA AGA TGC	TC TTT CAA CAC GCA GGA CAG
*IL-6*	GAC AGC CAC TCA CCT CTT CA	AGT GCC TCT TTG CTG CTT TC
*IL-8*	ATG ACT TCC AAG CTG GTG GCT	TTA TGA ATT CTC AGC CCT CTT CAA AAA
*IL-18*	GCT TTG GCC TTG GAA GAT GA	GAA GAT TCA AAT TGC ATC TTA T

## Supplementary Information

Fig. S1. Cytotoxicity of PYC in H292 cells. The graph bars indicate the cytotoxicity of PYC with H292 cells. Cells were seeded into 96 well plates, thus PYC (2.5-5-10-20-40 μg/ml) or AMP (2-Amino-4-methylpyridine; 5 μM) was treated. After treating the MTT solution of 5 μg/ml, plates were analyzed using a multifunctional plate reader (SPARK 10 M, Tecan, Switzerland) at OD 570 nm.

Fig. S2. Recovery of IκBα during infection with *P. aeruginosa* in H292 cells. H292 cells were seeded into 12 well plates and incubated for 24 h, The infection of *P. aeruginosa* (MOI 100) was performed up to 120 min after treatment with 20 μg/ml of PYC.
