# Supplementary material for: Pycnogenol reduces the expression of P. aeruginosa T3SS and inflammatory response in NCI-H292 cells
Source: J Microbiol. 2025 Sep 19;63(10):e2503004. doi: 10.71150/jm.2503004 (PMC13577061; doi:10.71150/jm.2503004)

Fig. S1. Cytotoxicity of PYC in H292 cells. The graph bars indicate the cytotoxicity of PYC with H292 cells. Cells were seeded into 96 well plates, thus PYC (2.5-5-10-20-40 μg/ml) or AMP (2-Amino-4-methylpyridine; 5 μM) was treated. After treating the MTT solution of 5 μg/ml, plates were analyzed using a multifunctional plate reader (SPARK 10 M, Tecan, Switzerland) at OD 570 nm.

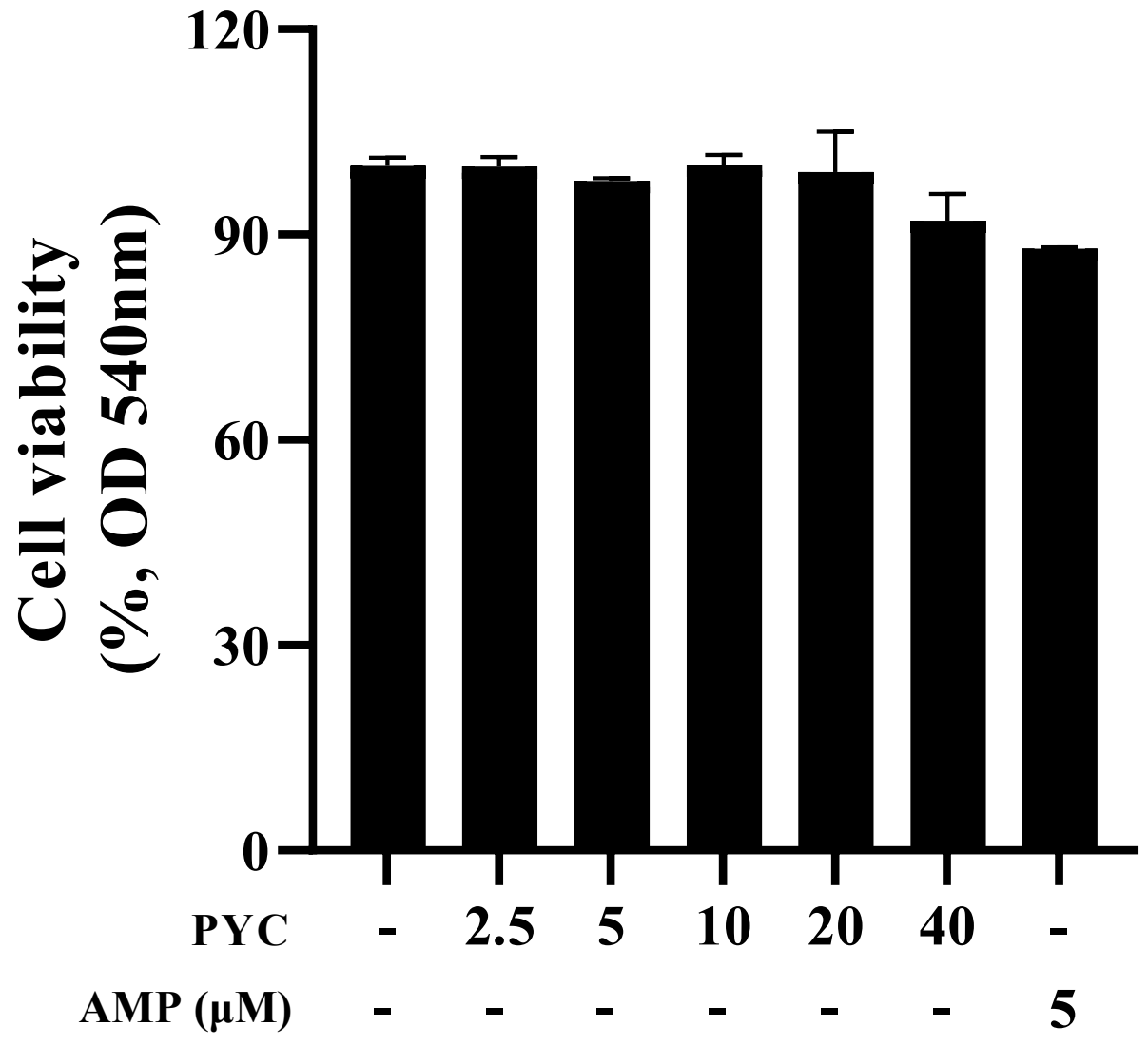

Supplement: Supplementary file 1 [file jm-2503004-Supplementary-Fig-S1.pdf]
