# Supplementary material for: Pycnogenol reduces the expression of P. aeruginosa T3SS and inflammatory response in NCI-H292 cells
Source: J Microbiol. 2025 Sep 19;63(10):e2503004. doi: 10.71150/jm.2503004 (PMC13577061; doi:10.71150/jm.2503004)

Fig. S2. Recovery of IκBα during infection with *P. aeruginosa* in H292 cells. H292 cells were seeded into 12 well plates and incubated for 24 h, The infection of *P. aeruginosa* (MOI 100) was performed up to 120 min after treatment with 20 μg/ml of PYC.

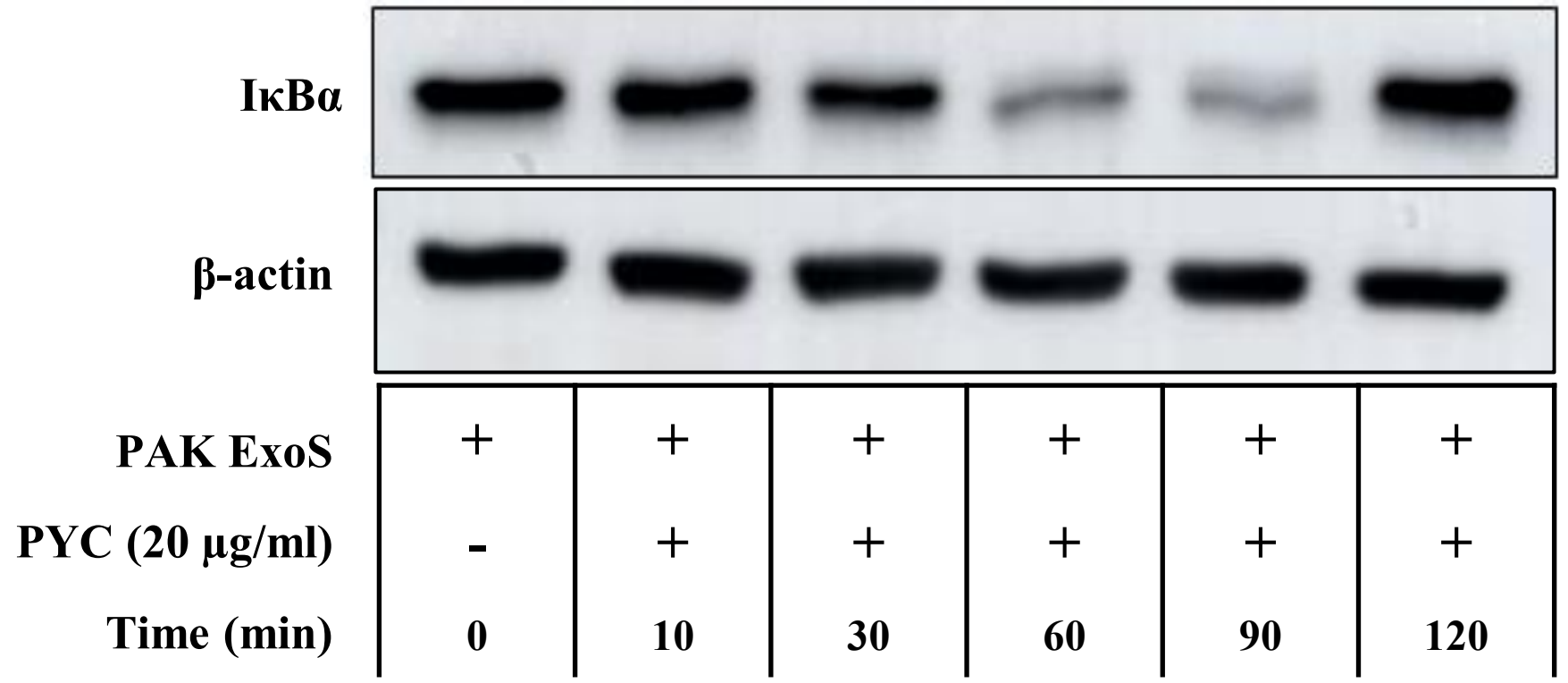

Supplement: Supplementary file 2 [file jm-2503004-Supplementary-Fig-S2.pdf]
